# Proteomics for Early Detection of Non-Muscle-Invasive Bladder Cancer: Clinically Useful Urine Protein Biomarkers

**DOI:** 10.3390/life12030395

**Published:** 2022-03-09

**Authors:** Jae-Hak Ahn, Chan-Koo Kang, Eun-Mee Kim, Ah-Ram Kim, Aram Kim

**Affiliations:** 1 Department of Urology, Konkuk University Medical Center, Konkuk University School of Medicine, Seoul 05030, Korea; 20180109@kuh.ac.kr; 2 Department of Advanced Convergence, Handong Global University, Pohang 37554, Gyeongbuk, Korea; ck@handong.edu; 3 School of Life Science, Handong Global University, Pohang 37554, Gyungbuk, Korea; 4 Department of Emergency Medical Technology, Korea Nazarene University, Cheonan 31172, Chungcheongnam-do, Korea; esther96@kornu.ac.kr

**Keywords:** bladder cancer, proteomics, urine, biomarker

## Abstract

Bladder cancer is the fourth most common cancer in men, and most cases are non-muscle-invasive. A high recurrence rate is a critical problem in non-muscle-invasive bladder cancer. The availability of few urine tests hinders the effective detection of superficial and small bladder tumors. Cystoscopy is the gold standard for diagnosis; however, it is associated with urinary tract infections, hematuria, and pain. Early detection is imperative, as intervention influences recurrence. Therefore, urinary biomarkers need to be developed to detect these bladder cancers. Recently, several protein candidates in the urine have been identified as biomarkers. In the present narrative review, the current status of the development of urinary protein biomarkers, including FDA-approved biomarkers, is summarized. Additionally, contemporary proteomic technologies, such as antibody-based methods, mass-spectrometry-based methods, and machine-learning-based diagnosis, are reported. Furthermore, new strategies for the rapid and correct profiling of potential biomarkers of bladder cancer in urine are introduced, along with their limitations. The advantages of urinary protein biomarkers and the development of several related technologies are highlighted in this review. Moreover, an in-depth understanding of the scientific background and available protocols in research and clinical applications of the surveillance of non-muscle bladder cancer is provided.

## 1. Introduction

### 1.1. Aim and Methods of the Study

Urinary biomarkers of superficial bladder cancers are still under-developed. In clinical settings, the few available options have poor sensitivity and specificity. Even though basic scientific advances are developing quickly, physicians still use conventional methods. Through this comprehensive review, physicians might gain new insights into new techniques in the scientific field and basic scientists may gain a deeper understanding of the urgent need related to these issues.

A literature search was performed on the PubMed electronic database using the following keywords: ‘proteomics bladder cancer’, ‘bladder cancer AND urinary biomarker’, ‘bladder cancer early detection AND proteomics’, and ‘bladder cancer recurrence AND proteomics’. The search targeted studies that were published in English within the past 20 years, except for a few articles.

### 1.2. Prevalence

Bladder cancer is the fifth most common malignancy worldwide, accounting for approximately 200,000 deaths annually [1]. Bladder cancers pose a high risk of recurrence and negatively impact patients’ quality of life. They account for 90% of all urothelial cancer cases. Most cases present with non-muscle-invasive bladder cancer (NMIBC). If diagnosed at early stages, NMIBC carries a favorable prognosis. However, NMIBC has a high recurrence rate of 80% in high-risk lesions and 50% in low-risk lesions. The 5-year survival rate of patients is 90% if the cancer is detected early. Therefore, early detection is critical, as intervention significantly influences the quality of life and overall survival of patients.

### 1.3. Grade and Stage of Bladder Cancer

Bladder cancer comprises 75% pure urothelial carcinoma and 25% “variant” histology, adding complexity to the diagnosis and treatment of this cancer [2]. It is classified into high- and low-grade diseases based on standardized histomorphological features as described by the World Health Organization. The tumor stage is defined as a measure of the depth of bladder wall invasion. Approximately 50% of NMIBC cases are low-grade, whereas most muscle-invasive or metastatic tumors are high-grade. Bladder tumors can be divided into papillary, solid, and mixed types. The papillary type is predominant, particularly in NMIBC [3].

### 1.4. Molecular Characteristics of Bladder Cancer

Phenotypes are associated with genetic alterations at the DNA and subsequent RNA expression levels and form several molecular subtypes with diagnostic, prognostic, and therapeutic implications. Initial investigations revealed the involvement of gene mutations in the development of bladder cancer, thus providing an insight into its response to conventional treatment and immunotherapy. Independent results from several studies have identified several common gene mutations in low-grade NMIBC; the genes involved are FGFR3, PIK3CA, STAG2, and the RTK/RAS/RAF pathway genes. High-grade MIBC/advanced disease involves mutations in *ERBB2*, *p53*, *RB1*, *MDM2*, *CDKN2A*, *KDM6A*, and *ARID1A* [4].

The molecular characteristics of bladder cancer can be grouped into luminal, basal, and squamous, and they determine the clinical response to neoadjuvant and adjuvant conventional chemotherapy, sensitivity to immunotherapy, and risk of progression and recurrence [5]. Basal bladder cancers are enriched with squamous and sarcomatoid characteristics that are expressed in the form of stemness and epithelial-to-mesenchymal transition; they are often invasive and metastatic at diagnosis. In addition, luminal types of bladder cancer are enriched with the papillary features and genetic mutations common in NMIBC, particularly FGFR3 mutations. Thus, luminal bladder cancers result from superficial cancers that progress to muscle invasion [6]. 

Based on the featured genetic changes, bladder cancers can be categorized into papillary and non-papillary groups. *FGFR3* gene mutations are the most critical in papillary tumors. Mutations in the major tumor suppressors TP53 and RB1 are important in non-papillary tumors. Both subtypes display high-frequency mutations in genes encoding the chromatin-modifying enzymes. However, mutations in the histone H3 lysine 4 (H3K4) methyltransferase KMT2D are more common in non-papillary cancers. In contrast, mutations in the histone H3 lysine 27 (H3K27) demethylase KDM6A are more common in papillary cancers. Mutations that activate the telomerase promoter and inactivate STAG2 are also commonly found in this category of papillary cancers [7].

### 1.5. Presentation and Diagnosis

The most common presentation of bladder cancer is gross hematuria. However, the patients may present microscopic hematuria (urinalysis showing three red blood cells per high-power field) and irritative voiding symptoms; the incidental discovery of a tumor upon imaging is also possible [8]. Urine cytology is widely used as a urinary biomarker. However, despite its high sensitivity in high-grade tumors and carcinoma in situ (CIS), the sensitivity and specificity of urine cytology remain poor in low-grade tumors [9]. 

### 1.6. The Urgent Need to Develop Urinary Biomarkers

To date, a few diagnostic urinary biomarkers have been developed for screening tumors and avoiding unnecessary cystoscopies. Some urinary biomarkers approved by the Food and Drug Administration (FDA) and the European Medicines Agency (EMA) are commercially available for urinary biomarker tests. Although the novel urinary tests showed relatively good outcomes in the MIBC setting, their application in the initial diagnosis of NMIBC is not well-supported by data. The diagnostic specificity of the Xpert Bladder Cancer Monitor test in patients with hematuria is promising but requires validation. Presently, the guidelines do not recommend routine urinary biomarker tests in the initial diagnosis. Hence, ongoing randomized trials should determine the benefits of using biomarkers in a large patient group (NCT03988309) [10]. A point-of-care urine test that general physicians can use to select patients for fast-track referral to the urologist should thus urgently be developed [11]. 

Urine cytology remains the most widely used non-invasive method for the diagnosis and surveillance of bladder cancer. This technique has displayed a specificity of almost 80–85%, but a low (40–50%) sensitivity has limited its application. The grading of urothelial carcinoma on urine samples becomes subjective and results in poor inter-observer variability [12]. 

**Figure 1:**
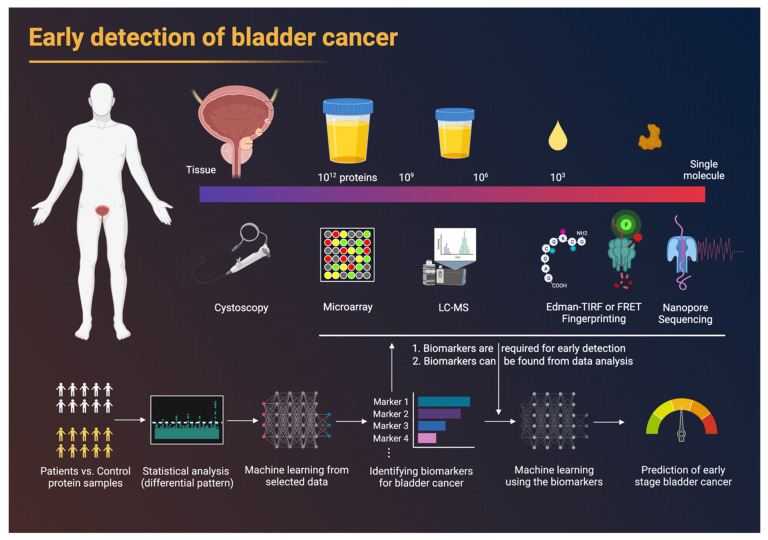
Development of proteomic tools for the early detection of bladder cancer.

Urinary biomarkers can play an essential role in the future of precision medicine, given the limitations of the currently available modalities, their specificity and sensitivity, and the need for invasive procedures to allow for surveillance. In addition to ensuring diagnostic accuracy, biomarkers must be reproducible, affordable, and easily implementable (Figure 1).

## 2. Proteomics

Proteins play a role in determining the identity of a cell [13]. Cell function can be affected by abnormal polypeptide sequences, altered protein expression, or abnormal post-translational modifications. They can be used as a biomarker for tumors as well [14]. The development of proteomics technologies has made it easier to identify protein biomarkers applied to tumor diagnosis. Current proteomics technologies can be categorized into antibody-, mass-spectrometry- (MS), and aptamer-based techniques [13]. In this chapter, we describe current proteomic technologies identifying bladder cancer biomarkers, including NMIBC. The following keywords were used to conduct a literature search on Google Scholar: ‘proteomics’, ‘bladder cancer AND proteomics’, ‘bladder cancer AND antibody AND proteomics’, ‘bladder cancer AND ELISA’, ‘bladder cancer AND MS’, ‘bladder cancer AND machine learning’, and ‘proteomics AND machine learning’. Apart from a few exceptions, the search focused on English-based journals from the past 20 years (Figure 1).

### 2.1. Antibody-Based Methods

The antibody-based methods, such as ELISA, protein microarray, and immunohistochemistry, have relatively simpler concepts than the MS- or aptamer-based techniques. When an antibody binds to a protein, it is detected as a dye or a fluorescence signal. This method can detect proteins at the 0.1–1 femtomolar (6 × 10^7^–6 × 10^8^ molecules, 1–10 pg/mL) level. The recently developed ultrasensitive ELISA can detect proteins at the zeptomolar (~600 molecules) level [15]. The amount of sample required for analysis is small compared to that used for MS, and no sample pre-treatment is required [16]. The simplicity of the antibody-based methods ensures their easy application in cost-effective diagnostic kits. Most FDA-approved biomarkers and cancer diagnosis kits, such as the NMP22 test kit, NMP22 BladderChek test, BTA TRAK, BTA stat, and UroVysion, use antibody-based methods [17,18,19]. Comparative studies on the early detection of NMIBC in body fluids, such as blood and urine, are currently underway [17,18,19,20,21,22,23,24,25,26,27,28,29,30]. However, it is difficult to identify novel biomarkers in protein mixtures using antibody-based methods. Although protein microarrays can measure hundreds to thousands of proteins simultaneously, they can only test those proteins for which antibodies are available and their fidelity is guaranteed. 

### 2.2. Mass-Spectrometry-Based Methods

Proteomics has advanced through developments in MS, which can identify, quantify, and explore protein biomarkers from protein pools with a high throughput. Additionally, the protein–protein interactions and post-translational modification [31] can be measured within a specific pool. Even de novo peptide sequencing can be performed, which is impossible when using antibody-based methods.

Recently, multiple biomarkers have been identified based on mass spectrometry, and protein differences in body fluid have been used to diagnose bladder cancer and classify NMIBC patients. Body fluids, such as blood serum [32,33], plasma [34], and urine [35,36,37], are the primary sources of non-invasive cancer biomarkers. Some proteins are much more abundant in some biological samples than others, leading to less abundant protein peaks that are almost invisible compared to the enormous peaks from the highly abundant ones. Therefore, there is inevitably a bias towards large amounts of protein in MS [13]. Several methods, such as nanoparticle-based methods, have been developed to remove the interfering proteins [32]. Both body fluids and tumor tissue samples can be used for MS. In most cases, freshly isolated tissue samples are used. The cryopreserved tumor tissue is treated with optimal cutting temperature compound (OCT) or formalin-fixation and paraffin-embedding (FFPE) methods for long-term storage, and preservatives, such as formalin, interfere with MS analysis. Sample preparation methods, such as filter-aided sample preparation (FASP), enable MS analysis using cryopreserved tissue samples [38].

To conduct MS analysis, it is often impractical to directly introduce intact sample proteins into the MS system. Identifying each protein in a sample based only on mass information is challenging because multiple proteins have similar molecular masses. Furthermore, owing to their size, proteins can be broken into many smaller pieces with trypsin or LysC. After the polypeptides are cleaved, the samples are subjected to isotope tags for absolute and relative quantification (iTRAQ), stable isotope labeling of amino acids in cell culture (SILAC), or tandem mass tagging (TMT). Recently, label-free quantification (LFQ) has become possible [31,38]. The tagged proteins are separated via liquid chromatography, high-pressure liquid chromatography, sodium dodecyl sulfate-polyacrylamide gel electrophoresis, or two-dimensional electrophoresis [34].

The molecules that are separated via chromatography or electrophoresis are charged via electrospray ionization (ESI) or matrix-assisted laser desorption/ionization (MALDI). These technologies are classified as soft ionization methods and are frequently used for proteomic analysis. They tend to fragment fewer large molecules than any other conventional ionization method (i.e., electron impact). MALDI produces far fewer multiple-charged ions, ensuring that more sample molecules are singly positively charged (+1) than with ESI. Ionized molecules can be measured by *m*/*z* detectors, such as magnetic sector MS, quadrupole MS, TOF, and ion traps. Proteins can be identified by comparing the measured *m*/*z* values with those in the protein database. 

It is beneficial to use tandem MS (MS/MS) to help with the resolution of MS. After measuring the first *m*/*z* (MS1), only molecules with the selected *m*/*z* (precursor ions) are resolved by collision and measured further (MS2). The MS2 *m*/*z* distribution of each polypeptide is unique because of collisions that cleave specific bonds (single bond breakage is most common). This allows for a complete analysis of the proteins. Using either data-dependent acquisition (DDA) or data-independent acquisition (DIA), the precursor ions can be passed to MS2. The top N molecules account for the largest proportion of distribution in the first MS; dynamic exclusion is the most common method used for DDA. As DDA only sends molecules with a specified *m*/*z* distribution to the second MS, a limited number of molecules can be measured in a short time, and the throughput is limited. However, all molecules within a certain *m*/*z* window are sent to MS2 in DDA. Furthermore, the *m*/*z* window is moved to analyze all molecules in the *m*/*z* distribution. Owing to the mixing of molecules in the *m*/*z* windows, the MS2 *m*/*z* distribution analysis becomes very complicated. A spectrum library is first created using the DDA method; DIA is then applied to resolve this problem. Analysis tools such as SWATH-MS interpret the MS2 *m*/*z* distribution. Recently, Data-dependent-independent acquisition (DDIA), which performs both DDA and DIA in one analysis, has been developed, thus simplifying the analysis [39]. 

### 2.3. Statistical Analysis and Machine-Learning-Based Diagnosis

To select biomarkers that are significantly detected in tumor samples, statistical analyses, including the t-test, analysis of variance (ANOVA), principal component analysis (PCA), Benjamini–Hochberg false discovery rate (FDR), and permutation-based FDR, are performed [14,31,33,34,38,40,41,42,43,44]. Previously, patients were classified using simple formulae for several biomarkers. Recently, a model that uses various biomarkers and machine learning techniques, such as support vector machine (SVM) and random forest, has been developed to diagnose cancer patients [40,42,45].

## 3. Urine Protein Biomarkers

A literature search was performed on the PubMed electronic database, using the following keywords: ‘proteomics bladder cancer’, ‘bladder cancer AND urinary biomarker’, ‘bladder cancer early detection AND proteomics’, and ‘bladder cancer recurrence AND proteomics’. The search targeted studies that were published in English-based journals within the past 20 years, except for a few articles.

### 3.1. Urinary Biomarker Tests for Diagnosis and Screening of NMIBC 

The urine is in direct contact with the tumor inside the bladder. Thus, the urinary proteome is a prominent diagnostic source because of the presence of specific proteins that represent the tumor molecular phenotype and directly reflect the bladder cancer biology. Moreover, the use of urinary biomarkers to detect BC is an attractive alternative in terms of both cost and convenience [46,47] (Table 1).

The United States FDA has currently approved six urinary biomarkers for the diagnosis and surveillance of bladder cancer. Nuclear matrix protein 22 (NMP22) is a non-chromatin protein that plays numerous roles in DNA replication and gene expression. It can function as a urinary biomarker of urothelial cell death. In urothelial tumors, the levels of NMPs are high because of cell turnover caused by tumor apoptosis [17,19]. In a systematic review of 23 studies and a quantitative meta-analysis of 19 studies on the detection of bladder cancer, the sensitivity of NMP22 was found to be 52–59%, and the specificity was 87–89%, with an area under the ROC curve (AUC) of 0.83. The mean sensitivities for Ta, T1, ≥T2, and Tis were 13.68%, 29.49%, and 74.03%, respectively, and for G1, G2, and G3 diseases they were 34.62%, 44.16%, and 56.25%, respectively [48].

Bladder tumor antigen (BTA) is another biomarker approved by the FDA. The BTA test detects the human complement factor H-related protein (hCFHrp), which is produced to protect cells from complement activation and is found in bladder cancer cell lines. In a meta-analysis of 13 studies (consisting of 3462 patients with bladder cancer) using the BTA STAT test, the sensitivity of BTA was 64–69% and the specificity was 73–77% [49], whereas the BTA TRAK test exhibited a sensitivity of 62–71% and a specificity of 45–81% [50]. The sensitivity of BTA was positively correlated with the increasing tumor grade in bladder cancer, similar to the trend observed for other biomarkers. 

The ImmunoCyt/uCyt^+^ test is an immunocytochemical test that utilizes three fluorescent monoclonal antibodies (M344, LDQ10, and 19A211) to detect the carcinoembryonic antigen and two mucins in exfoliated urothelial cells in voided urine. In seven separate studies consisting of 1602 patients with bladder cancer, the ImmunoCyt test exhibited a sensitivity of 72.5% (95% CI, 68.3–76.5%) and a specificity of 65.7% (95% CI, 62.9–68.5%) [51].

UroVysion is a molecular test that employs the fluorescence in situ hybridization (FISH) probe to detect the aneuploidy of chromosomes 3, 7, and 17 and the loss of the *p16* gene at the 9p21 locus; these are some of the genetic abnormalities observed in bladder cancer. The pooled results from a meta-analysis revealed a sensitivity of 72% and a specificity of 83% of UroVysion for the detection of bladder cancer [52]. 

The UBC^®^ Rapid Test measures the soluble fragments of cytokeratins 8 and 18 in the urine. Urine samples were collected from 111 patients with bladder cancer and 133 clinical controls without urological disease. In a study, the UBC^®^ Rapid Test displayed a sensitivity of 56% and a specificity of 96% for the detection of bladder cancer [53]. In a multi-center study focusing on non-muscle-invasive high-grade bladder cancer, the levels of cytokeratins in the urine were higher in patients with bladder cancer than in the healthy controls without a history of bladder cancer. The sensitivity was 38.8% for non-muscle-invasive low-grade cancers, 75.0% for non-muscle-invasive high-grade cancers, and 68.3% for muscle-invasive high-grade bladder cancers; the specificity was 93.8% for all calculations [29].

The proteolytic region of cytokeratin-19, referred to as Cyfra 21-1, is a soluble molecule present in the serum and other body fluids and is considered a tumor marker in several neoplastic diseases [26]. In a study analyzing urine samples from 325 patients, the Cyfra 21-1 marker showed a sensitivity of 79.3% and a specificity of 88.6% in individuals presenting with hematuria or irritative voiding symptoms. The mean urine Cyfra 21-1 levels in patients with grade 1, 2, and 3 tumors were 12-fold, 28-fold, and 37-fold higher than those in healthy controls, respectively [23].

Urothelial bladder carcinoma 1 (BLCA-1) and urothelial bladder carcinoma 4 (BLCA-4) are nuclear transcription factors identified during the early stages of bladder cancer. Their levels increase during the early development of bladder cancer and could potentially be used as biomarkers at an early stage. BLCA-1 has a sensitivity of 80% and a specificity of 87% for the detection of bladder cancer [54]. The urine BLCA-4 levels in patients with bladder cancer were significantly higher than those in the control group in a previous study [55]. In another study, BLCA-4 was independently validated with a sensitivity of 93% and a specificity of 97% [56].

CellDetect is a histochemical staining technique that utilizes color and morphology to discriminate between malignant and benign cells based on differences in metabolic signatures. In two studies, CellDetect exhibited a higher sensitivity for low-grade tumors than urine cytology (82% vs. 59%) and a similar specificity (86% vs. 94%) [57,58]. 

Hyaluronidase can improve cell proliferation and motility via HA [59]. HA levels are elevated in the urine of patients with bladder cancer. In a study analyzing 139 specimens, urinary hyaluronidase levels were 5–8-fold higher in patients with G2/G3 bladder cancer than in healthy controls [60]. Studies have shown that the sensitivity and specificity of hyaluronidase levels for the detection of bladder cancer range from 87–100% and 89–98%, respectively [61,62,63,64].

sFas is an anti-apoptotic protein released by bladder cancer cells to protect themselves from anti-tumor activity. In a study examining urinary sFas concentrations in 74 controls and 117 cases of TCC, urinary sFas concentrations were found to be significantly higher in bladder cancer patients than in normal controls. Urinary sFas levels have a sensitivity and specificity of 88.03% and 89.19%, respectively, for the detection of bladder cancer [65]. Urinary sFas levels are significantly higher in patients with NMIBC than in those without NMIBC (*p* = 0.000). However, higher levels are associated with a higher risk of recurrence [66].

Survivin is an inhibitor of apoptosis that is overexpressed in many malignancies but is rarely detected in normal tissues. Functionally, survivin inhibits apoptosis and promotes cell proliferation and angiogenesis [67]. Survivin levels are associated with bladder cancer and higher tumor grades. In a study, urinary survivin was detected in all 46 patients with bladder cancer but not in 32 of 35 samples of patients treated for bladder cancer who had negative cystoscopy results. In another study, survivin levels in both healthy controls and patients with other genitourinary cancers were normal [68]. A study investigated 118 urine samples from 24 patients with bladder cancer, 50 with a bladder cancer history, 68 not known to harbor bladder cancer, and 55 with hematuria. Survivin expression in the urine samples of urological patients was detected with a sensitivity and specificity of 79% and 93%, respectively [69]. 

The ADXBLADDER test is a urine test that detects mini chromosome maintenance 5 (MCM5), which is present in urine sediment and indicates the presence of a bladder tumor. The test is superior to urine cytology for detecting NMIBC recurrence [30]. The DNA replication licensing factor MCM5 is not influenced by infection or inflammation, unlike other biomarkers. In July 2020, the UK National Health Service approved the use of the ADXBLADDER test to aid in the diagnosis and surveillance of bladder cancer. The test has an overall sensitivity and specificity of 45–73% and 70–73%, respectively [30,70].

The URO17 urinary biomarker is an immune biomarker that binds to the oncoprotein keratin 17. In a discovery cohort comprising 81 samples, the URO17 immunocytochemistry (ICC) test for biopsy-confirmed urothelial carcinoma exhibited 97% sensitivity; the sensitivity was 86% in the validation cohort with 98 samples [71]. URO17 could detect both low- and high-grade cancers in patients presenting hematuria and exhibited a specificity of 96% and 92.7% in recurrent and newly diagnosed patients, respectively; the AUC was 0.90 [72,73].

Apo-A1 is the primary protein component of high-density lipoproteins and promotes tumor angiogenesis through kinase activation [74,75]. In a study evaluating the potential of Apo-A1 as a biomarker of bladder cancer, two-dimensional electrophoresis and subsequent MS were used to demonstrate the increased expression of Apo-A1, which was confirmed by Western blot results. A study of 379 urine samples showed a sensitivity and specificity of 89.2% and 84.6%, respectively, of this biomarker (PMID: 24661883). In other such studies, Apo-A1 involvement was independently validated in bladder cancer, with 92–95% sensitivity and 85–92% specificity [76,77]. 

The biomarker panel approach for bladder cancer is challenging because the practical value of individual biomarkers remains unproven. The combination of disease-specific biomarkers can improve diagnostic efficacy. A study of voided urine samples from 127 patients analyzed various multivariate combinations of 14 biomarkers (IL-8, MMP-9, MMP-10, SDC1, CCL19, PAI-1, CD44, VEGF, ANG, CA9, A1AT, OPN, PTX3, and APOE) and identified a biomarker panel that could outperform the current urinary biomarkers. The biomarker panel included ANG, apolipoprotein E (APOE), CA-9, interleukin-8 (IL-8), MMP, MMP10, plasminogen activator inhibitor 1 (PAI-1), and VEGF and achieved a sensitivity of 92% and a specificity of 97%. However, these biomarkers have not yet been independently validated [59,78].

The present question revolves around improving the sensitivity and specificity of urinary biomarkers, especially in low-grade bladder cancers. In the above-mentioned tests, false-positive results are common because urinary biomarkers are also released during hematuria, inflammation, urolithiasis, recent instrumentation, other genitourinary malignancies, intravesical Bacillus Calmette–Guerin (BCG), or infection [49,79,80,81,82,83,84,85,86].

### 3.2. Diagnostic Tests for Determining the Accuracy of FDA-Approved Biomarkers in Detecting NMIBC Recurrence

#### 3.2.1. NMP22

In a prospective study (*n* = 156) to determine the utility of urinary NMP22 in monitoring superficial bladder cancer after transurethral resection, the sensitivity of NMP22 was 48.8%, with an ideal cut-off value (5.0 U/mL) [87] (Table 2).

#### 3.2.2. BTA

In a cohort of 26 patients from whom 111 urine specimens were collected, the BTA test exhibited a sensitivity of 60.7% and a specificity of 74.1%. The accuracy was 64%, with a positive predictive value of 87.9% and a negative predictive value of 37.7% for detecting bladder carcinoma recurrence [63].

#### 3.2.3. ImmunoCyt

In a meta-analysis of 57 studies, except case–control studies, ImmunoCyt for surveillance exhibited a sensitivity of 75% (Cl, 64–83%) and a specificity of 76% (Cl, 70–81%) [80]. Another prospective study involving 942 patients demonstrated that the sensitivity of ImmunoCyt for detecting the recurrence of grade 1, 2, and 3 tumors was 79.3%, 84.1%, and 92.1%, respectively [88].

#### 3.2.4. UroVysion (Multi-Target Fluorescence In Situ Hybridization)

The objective of this study was to evaluate the diagnostic value of chromosomal analysis using fluorescence in situ hybridization (FISH) in predicting the recurrence of urothelial carcinoma after transurethral resection. Recurrence was observed in 39% of the 50 patients with a positive FISH test, but only 21% of the 88 patients showed negative FISH tests [89].

## 4. New Technologies

Liquid chromatography–mass spectrometry (LC/MS) and antibody-based microarrays are the key technologies in contemporary proteomics-based research. These techniques, in combination with various labeling techniques, have facilitated large-scale quantitative protein analysis for biomarker discovery. However, despite their significant contributions, LC/MS-based and antibody-based technologies are not high-throughput technologies for the rapid profiling of potential biomarkers for various diseases, including bladder cancers.

LC/MS technology, the gold standard for current proteomics research, analyzes only the *m*/*z* peaks of protein fragments. The analysis of complex protein samples with many similar *m*/*z* characteristics is complicated. To reduce the complexity of the sample, it is pretreated and proteins are separated using 2D gel electrophoresis, which results in a long cleaning time between experiments. Additionally, attomolar to femtomolar (fM) concentrations of molecules (−10^6^–10^9^ molecules) are required to identify and quantify each protein [90].

Antibody-based microarrays have an even lower sensitivity (fM to pM range; −10^9^–10^12^ molecules) [91]. Another problem is the limited availability of antibodies for the total number of human proteins. These limitations increase the labor and time required to generate proteomics data, making it challenging to discover and analyze biomarkers in small amounts of blood and urine samples.

For genomics and transcriptomics research, second-generation (also known as massively parallel sequencing) and third-generation sequencing (Single-molecule, long-read sequencing) technologies have been developed to rapidly and accurately identify and quantify DNA/RNA molecules in the test samples. Next-generation DNA sequencing can be commercialized rapidly because only four types of nucleotides need to be sequenced. Moreover, template-directed amplification technology, such as PCR, makes it possible to analyze tiny amounts of DNA samples and easily label the DNA being amplified with fluorescent tags.

Unfortunately, no method with a similar scale and sensitivity to that of next-generation DNA sequencing exists that can identify and quantify specific proteins in complex mixtures [92]. There were two significant technical limitations to developing high-throughput, high-sensitivity protein sequencing methods in the abovementioned study. First, template-directed protein amplification was not performed. Second, as specific proteins consist of 20 amino acids, it was difficult to precisely read their sequences via optical or electrochemical measurements.

Novel methods have recently been developed to overcome these limitations. The first strategy is the distribution of a few types of amino acids labeled with different fluorescent tags instead of reading every protein sequence. In 2018, a research team succeeded in sequentially reading the cysteine (C) and lysine (K) residues in two different peptides using fluorescence resonance energy transfer (FRET) [93]. The immobilized ClpXP protease functions as a protein scanner and recognizes a specific sequence attached to the end of each peptide. It then linearizes the peptide and draws it into its interval cavity for degradation. FRET occurs when each of the acceptor fluorophore-labeled CK amino acids is pulled into the donor fluorophore-labeled ClpXP, creating a unique CK read for each peptide. The readout of the CK sequences (called protein fingerprints) is then used to identify the protein of interest using a protein database. A computational analysis showed that if additional parameters, such as the distance between cysteines and lysines, were considered, the single-molecule protein fingerprinting method could accurately identify a significant percentage (>70–80%) of proteins, even when considering the high error rates (20–30%) [94].

Another important approach has recently been reported [95]. It involves a nanopore-based single-molecule protein sequence that reads the entire sequence of individual protein molecules. Since its first commercial release in 2014 as a single-molecule DNA sequencer, nanopore-based DNA sequencing has undergone remarkable advancements. Efforts to utilize nanopore technology for protein sequencing are ongoing, following the success of single-molecule DNA sequencing using biological nanopores. In 2021, a research team succeeded in the multiple rereading of single proteins at a single-amino-acid resolution using nanopores. In their study, they succeeded in reading up to the first 25 amino acid residues of the protein. The nanopore-based sequencing method exhibits the capability to “rewind” peptide reads. It obtains numerous independent reads of the same molecule, yielding an error rate of <10^−6^ in single amino acid variant identification [96]. Another accomplishment of this study was that it achieved a reasonable sequencing speed. If the speed of molecules passing through the nanopore is too high, it is difficult to measure and interpret electrical signals. Similarly, if the speed is too low, the efficiency of sequencing decreases. This system was designed to read approximately 80 amino acids per second, enabling sufficient signal reading and a fast sequencing speed.

Its goal is the same as that of a third-generation DNA sequencing technology; in principle, it can identify a single protein molecule in a sample. Because a massively parallel nanopore fabrication technique already exists, the success of protein sequencing through nanopores will lead to a breakthrough in proteomics research.

With the remarkable advancement of next-generation protein sequencing technology, it will soon be possible to analyze whole proteomes in trace samples, eventually generating a vast amount of single-cell-level proteome data. AI-based technology will play a key role in identifying meaningful patterns and information, such as biomarkers of various diseases, from large amounts of data. In addition, machine learning, combined with proteomics data, will allow a more accurate early diagnosis, differential diagnosis, and prognosis of the future clinical disease course [97] (Figure 1). 

## 5. Conclusions

We explored the promising role of urine protein biomarkers in the screening and diagnosis of superficial bladder cancer. A new diagnostic method using urine biomarkers will enable the early detection of bladder cancer without the traditional invasive cystoscopy. However, currently reported biomarkers associated with the development of bladder cancer are not sufficient for use in clinical settings. Although several urine biomarkers have been identified, they show modest levels of sensitivity and specificity and only a few urinary biomarkers have been approved by the US FDA so far.

Recent advances in proteomics and computational biology are accelerating our understanding of the biology and pathology of bladder cancer. With these new advances in urine-based non-invasive technology, superficial bladder cancer will be detected much earlier than with conventional methods. Artificial intelligence trained with the massive clinical proteomics data of randomized controlled trials will dramatically increase the accuracy of non-invasive bladder cancer tests.

## Figures and Tables

**Table 1:** Non-FDA-approved urine tests.

Biomarker	Method	Sensitivity (%)	Specificity (%)
UBC	ELISA Immunoradiometric assay	64.4	80.3
CYFRA21-1	ELISA	82	80
BLCA-1	ELISA	80	87
BLCA-4	ELISA	93	97
CellDetect	Immunostaining	84	70
Hyaluronic acid	ELISA RT-qPCR	87–100	89–98
sFas	ELISA	51.2	85.9
Survivin	Bio-dot test	79	93
MCM5-ADXBLADDER	ELISA	51.9	66.4
URO17	Immunocytochemistry	97	AUC: 90
Apo-A1	ELISA	83.7–95	85–97
ANG, APOE, CA9, IL-8, MMP9, MMP10, PAI-1, and VEGF	ELISA	92	97

**Table 2:** FDA-approved urine tests.

Test	Biomarker	Method	Sensitivity (%)	Specificity (%)
NMP22	NMP-22	Sandwich immunoassay	52–59	87–89
BTA stat^®^	Complement factor H-related protein	Colorimetric immunoassay	64–69	73–77
BTA TRAK^®^	Complement factor H-related protein	Sandwich immunoassay	62–71	45–81
ImmunoCyt^TM^	Carcinoembryonic antigen and two mucins (M344, LDQ10, and 19A11)	Immunofluorescence cytology	78	78
UroVysion^TM^	Aneuploidy of chromosomes 3, 7, and 17 and loss of the 9p21 locus	Multi-target FISH	63 (30–86)	87 (63–95)

## Data Availability

Not applicable.
